# Genotype-Epigenotype Interaction at the *IGF2* DMR

**DOI:** 10.3390/genes6030777

**Published:** 2015-08-28

**Authors:** Susan K. Murphy, Erin Erginer, Zhiqing Huang, Zachary Visco, Cathrine Hoyo

**Affiliations:** 1 Department of Obstetrics and Gynecology, Division of Gynecologic Oncology, Duke University Medical Center, Box 91012, B223 LSRC Building, Durham, NC 27708, USA; E-Mails: erin.erginer@gmail.com (E.E.); zhiqing.huang@duke.edu (Z.H.); zachary.visco@duke.edu (Z.V.); 2 Department of Biological Sciences, North Carolina State University, Raleigh, NC 27695, USA; E-Mail: choyo@ncsu.edu

**Keywords:** *Insulin-like Growth Factor II*, differentially methylated region, polymorphism, hypomethylation, CpG dinucleotide, imprinted gene

## Abstract

Paternally expressed Insulin-like Growth Factor II (*IGF2*) encodes a gene whose protein product functions as a potent growth mitogen. Overexpression of IGF2 has been implicated in a wide number of disorders and diseases. *IGF2* is regulated in part by differential methylation of the two parentally derived alleles. The differentially methylated region (DMR) located upstream of the imprinted promoters of *IGF2* exhibits plasticity under environmental stress and is hypomethylated in several types of cancer. Through bisulfite pyrosequencing and confirmation by nucleotide sequencing, we discovered a CpG to CpC transversion that results in hypomethylation of one of the three CpGs comprising this DMR. The presence of the polymorphism introduces a genetic rather than an environmentally-driven epigenetic source of hypomethylation that is additive to non-genetic sources.

## 1. Introduction

Numerous studies have focused on analysis of DNA methylation at a region located upstream of the three major imprinted promoters of *IGF2* in humans. This region is one of the differentially methylated regions (DMRs) contributing to the regulation of Insulin-like Growth Factor II (*IGF2*), an imprinted gene that is expressed from the paternally-derived chromosome. *IGF2* encodes for a small protein that is part of the insulin family and functions as a signaling molecule through binding to the IGF1 and insulin receptors [1]. IGF2 protein also binds to the membrane-bound and soluble forms of the IGF2 receptor, but this leads to internalization and degradation of IGF2 in the lysosomes. IGF2 has been shown to be deregulated in neurodevelopmental disorders [2,3,4], obesity and cancer [1,5], and is also known to have a critical role in memory consolidation in the brain [6,7,8,9].

DNA methylation occurs at the 5'-carbon position of the cytosine ring at cytosines that are followed by guanines (CG dinucleotides) in the DNA sequence. DNA methyltransferase (DNMT) enzymes recognize the palindromic 5'-CG-3' dinucleotide and are able to establish methylation *de novo* (DNMT3A and DNMT3B) or copy the methylation pattern of the parent DNA strand onto newly replicated DNA (DNMT1) (for review, please see [10,11]). Most CG dinucleotides throughout the genome are methylated. For genes subjected to parent-of-origin-dependent, monoallelic expression, CG dinucleotides are methylated on one parental strand and the same CG dinucleotide sequence on the other parental strand is unmethylated. This pattern of differential methylation is established during gametogenesis. For imprinted *IGF2*, these regulatory methylation marks are established during spermatogenesis, while the same sequences in the oocyte are unmethylated.

The multifaceted effects of IGF2 have led many, including our group, to study how the methylation status of this DMR, hereafter referred to as the *IGF2* DMR, varies in disease states [12,13,14,15], as well as how methylation of this region is influenced by the *in utero* environment [16,17,18,19,20,21,22,23]. The first reports of methylation plasticity of the *IGF2* DMR came from studies of individuals with colon cancer. In these studies, hypomethylation of the three CpG sites comprising the DMR was reported [24]. This was also detectable in peripheral blood of the individuals with colon cancer and in 10% of an otherwise healthy control population [25]. This same region has also been the focus of studies of individuals conceived during the Hunger Winter in the Netherlands at the end of World War II. Hypomethylation of this DMR was associated with exposure to caloric restriction *in utero* and this was evident decades later [26]. We have also found that methylation of this region in newborns is vulnerable to the effects of *in utero* exposure to cigarette smoking [18] and paternal obesity [27]. Hypomethylation has also been associated with increased circulating levels of IGF2 protein and increased birth weight [20], and conversely, low birth weight for children born to mothers who are depressed during pregnancy [17].

Herein we report the identification a G > C polymorphism that directly affects one of the three CpG sites that comprise the *IGF2* DMR, resulting in a CpC dinucleotide. The presence of this genetic polymorphism necessarily results in loss of methylation at this position, and thus overall decreased methylation of this DMR, through genetic ablation of the DNA methyltransferase recognition sequence at this CpG dinucleotide. Furthermore, the polymorphism appears to be more common in individuals with African heritage. These findings have implications for the methylation status of this region and consequent effects on *IGF2* expression and imprinting.

## 2. Materials and Methods

### Human Subjects

Cervical Cancer Screening Cohort, Moshi, TZ: The study population has been previously described, along with eligibility criteria and recruitment procedures [28]. Briefly, 249 participants were recruited of 251 approached between November 2008 and March 2009 from the Reproductive Health Clinic (RHC) at Kilimanjaro Christian Medical Center (KCMC), a Cervical Cancer prevention clinic. Eligibility criteria included ≥18 years of age, no prior history of an abnormal Pap test and willingness to participate. Invasive cervical cancer patients were also 18 years or older and were referred for colposcopic directed evaluations. Methylation data for the *IGF2* DMR was generated for 166 of these women [13]. All subjects gave their informed consent for inclusion before they participated in this study. This study was conducted in accordance with the Declaration of Helsinki and the protocol was approved by the Research Ethics Board at KCMC and by the Duke University Institutional Review Board (Pro00008813).

The Newborn Epigenetics STudy (NEST), Durham, NC, USA: Study participants were enrolled between 2005–2009 and 2009–2011 as part of two multiethnic birth cohorts designed to identify the effects of early exposures on epigenetic profiles and phenotypic outcomes. These studies were conducted in accordance with the Declaration of Helsinki and approved by the Duke University Institutional Review Board (Pro00014548). Written consent was obtained from all mothers participating as study subjects prior to their inclusion in the study. Details regarding identification and enrollment procedures have been described elsewhere [19,29]. A total of 3646 pregnant women were approached and 2587 consented to participate in NEST. Methylation data for the *IGF2* DMR was completed for 1104 newborns.

*Bisulfite pyrosequencing.* Genomic DNA was extracted from cervical scrapes, biopsies and invasive cervical cancer specimens (described in [30]) using Gentra Puregene Reagents (Qiagen, Valencia, CA, USA). Genomic DNA was extracted from umbilical cord blood in the NEST cohort also using Gentra Puregene reagents. The *IGF2* DMR was analyzed by bisulfite pyrosequencing. Bisulfite modification of 800 ng genomic DNA was performed as previously described [31] or using the Zymo EZ DNA Methylation^TM^ Kit according to the manufacturer’s recommendations (Zymo Research; Irvine, CA, USA). Pyrosequencing assays were designed using PSQ Assay Design Software and reactions were run on a Pyromark Q96 MD Pyrosequencer (Qiagen). The percent methylation for each of the CpGs within the target sequence was calculated using PyroQ CpG Software (Qiagen).

The region analyzed for the *IGF2* DMR includes three CpG dinucleotides upstream of exon 3 (chr 11p15.5; CpG site 1: 2,169,519; CpG site 2: 2,169,516; and CpG site 3: 2,169,499; NCBI Human Genome Build 37/hg19) [24]. PCR and pyrosequencing primers and conditions were as described [17,19]. Assay validation was performed by analysis of defined ratios of plasmids that contain inserts derived from the bisulfite modified version of the methylated and unmethylated sequences, as previously described [32].

*Nucleotide sequencing.* Genomic DNA underwent PCR using primers F: 5'-TTT CCC TGG GAA TGC TCA TTC-3' and R: 5'-TTC TGT TGG ACA GGC TGC CC-3'. Genomic DNA (20 ng) was amplified in a 12.5 µL PCR reaction volume using Hotstar Taq DNA polymerase (Qiagen). Thermocycler conditions were as follows: 95 °C for 15 min followed by 35 cycles of 95 °C for 30 s, 67 °C for 30 s and 72 °C for 30 s, and a final extension at 72 °C for 10 min. The amplicons were resolved on 2% agarose gels stained with ethidium bromide, the bands excised and amplicons separated from the agarose using GenElute Agarose Spin Columns (Sigma-Aldrich Corp.; St. Louis, MO, USA). Sequencing reactions were performed with BigDye Terminator reagents (Life Technologies; Grand Island, NY, USA). The product was sequenced using primer 5'-ATG CAT GAA GTT TTT CTC TG-3' at the Duke Genome Sequencing and Analysis Core Facility.

*Statistical analysis.* The potential over-representation of the polymorphism by race or by disease status was analyzed using Fisher’s exact tests, with a *p* value < 0.05 considered significant.

## 3. Results

In prior published work, we have reported on methylation of the *IGF2* DMR in two distinct cohorts. The first cohort analyzed included 1104 newborn participants of our Newborn Epigenetics Study from Durham, NC, using umbilical cord blood [17,19]. The second cohort analyzed were adult women from Moshi, Tanzania who provided cervical scrapes as part of their participation in a study of the progression of cervical intraepithelial neoplasia [28]. As part of our analysis of DNA methylation at regulatory regions of imprinted genes for these two studies, we used bisulfite pyrosequencing to quantify methylation of the same three CpG sites within the *IGF2* DMR as first defined by Cui *et al.* [24]. We have previously validated the performance of this assay and shown the ability to discriminate 5% differences in methylation across the full range of possible values, using defined mixtures of methylated and unmethylated DNAs [17].

The pyrosequencing software uses information from a pre-defined sequence provided from user input during the assay setup that determines the order of nucleotides to be added, one at a time, during the sequencing reaction. The pre-defined sequence allows for detection of methylation status at CpG dinucleotides by step-wise injection of “T” (unmethylated) and then “C” (methylated). It then calculates the proportion of C from the peak height obtained relative to the total peak heights obtained for the T and C combined at that particular cytosine position, prior to injection of the guanine nucleotide at the following step that completes the CpG dinucleotide sequence. Mononucleotide runs, especially Ts, are common due to the conversion of non-CpG cytosines to uracils, then to thymines following PCR. Such runs are accounted for by the height of the peak obtained. The peak height should be divisible by the number of same nucleotides in the run to yield a number that is equivalent to that obtained for the peak height of a single nucleotide.

**Figure 1:**
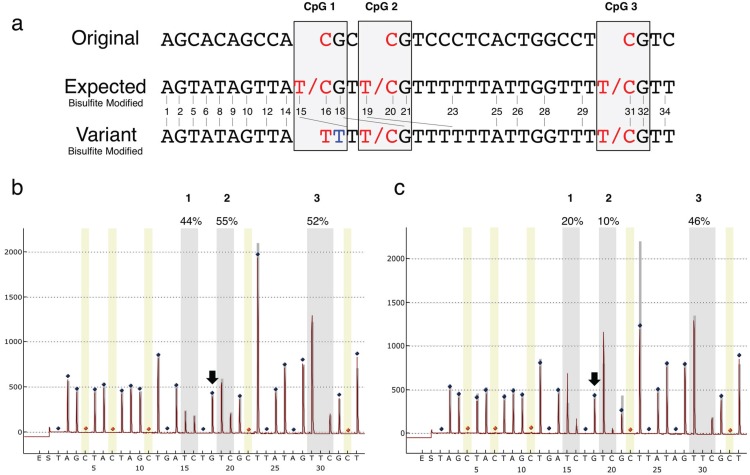
(**a**) The original sequence of interest for the *IGF2* DMR region and the expected sequence from the bisulfite modified DNA and pyrosequencing, alongside the sequence for the variant allele. The three CpG dinucleotides are labeled; (**b**) Pyrogram obtained for an individual with wild type alleles; (**c**) Pyrogram from an individual with the variant allele, showing low methylation of CpG position 1 and a much higher than expected incorporation of T nucleotides at CpG 2 (position 19) due to the creation of a 3–4 base mononucleotide stretch of T residues. The number of T residues incorporated depends on the methylation status of the second CpG cytosine. This leads to premature incorporation of the downstream T nucleotides at position 19, early incorporation of the downstream G at position 21 instead at position 18 on the variant allele and as consequence only half the anticipated G height at the subsequent G at position 21, and lower than expected peak height for the Ts at position 23 since those on the variant allele had been incorporated at position 19. This also results in inaccuracy for the methylation status of the second CpG (site 20). The % methylation measured is indicated above each CpG cytosine. CpG cytosine positions are indicated by the grey vertical bars. Blue diamonds, non-variable reference peaks; orange diamonds with yellow background bar, bisulfite treatment control and reference peak; narrow grey bars behind peaks, theoretical histogram for the sequence to analyze. Methylation data for the *IGF2* DMR was generated for *n* = 166 cervical cancer study specimens, of which *n* = 19 had the variant allele, and *n* = 1104 NEST cohort specimens, of which *n* = 50 had the variant allele.

At the end of each run, the Pyromark CpG software assigns a “pass”, “check quality” or “fail” descriptor for each sample. Whereas “pass” means that the sequence obtained and measured is within the limits of what might be expected given the sequence being analyzed. “Fail” means that a substantial problem has occurred except in the case of “no template” controls that are scored as “failed” runs when they have worked appropriately. A “check quality” score means there is a problem, but it may be minor or of no consequence and the software leaves it up to the user for interpretation. During study of the two cohorts described, we incidentally noted that there were multiple samples among our cohorts that were scored as “failed” runs by the PyroMark CpG software. We attempted to repeat the pyrosequencing reactions but to no avail. Since DNA quality can affect the ability to successfully perform pyrosequencing, we initially attributed failure of these runs to poor DNA quality and excluded these specimens from our original analyses. However, upon later visual inspection of the raw data, it was apparent that all of the “failed” runs were similar at the first CpG position of the *IGF2* DMR and had a “failed” reference sequence at several positions downstream. These were followed by resumption of normal sequence patterns to the end of the sequence analyzed (Figure 1). The T incorporation at position 15 (Figure 1c) was much greater than expected, even if the cytosine had been unmethylated in the original sequence. This also led to problems with the second CpG site in the sequence (position 19), due to sequencing of the two alleles being out of sync until reaching an A residue (position 25) located a few bases prior to the 3rd CpG site (position 31), which shows the expected pattern of methylation in these individuals. We hypothesized that this pattern might result from the presence of a polymorphic nucleotide variant in the sequence.

**Figure 2:**
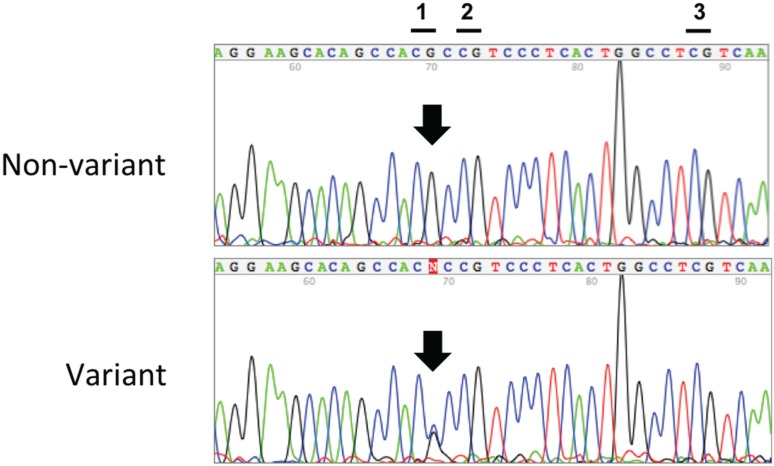
Representative nucleotide sequencing of the non-bisulfite modified genomic DNA for *n* = 14 variant cervical tissue specimens, *n* = 2 WT cervical cancer specimens and *n* = 6 cord blood specimens. The non-variant (**top**) and variant (**bottom**) nucleotide sequences of the *IGF2* DMR region are shown. The three CpG sites comprising this DMR are designated at the top and the position of SNP G > C rs116779517 is indicated by the arrow.

Nucleotide sequencing of the unmodified genomic DNA indeed confirmed the presence of a G/C transversion polymorphism at CpG position 1, which changes the CpG dinucleotide to a CpC dinucleotide on the affected allele (Figure 2). This SNP, subsequently identified as SNP rs116779517 (dbSNP 135), abolishes the 5'-CG-3' recognition sequence for the DNA methyltransferase enzymes—and thus the ability to methylate this cytosine. The decreased methylation observed at the second CpG dinucleotide was an artifact due entirely to the pyrosequencing method itself, whereby incorporation of nucleotides at the second CpG site occurred prematurely at the position of the first CpG site, rather than to any additional sequence variants being present, as described above.

We observed this polymorphism in 4.5% (50/1104 individuals; minor allele frequency of 0.02) of our newborn cohort (Table 1) as well as in 11.4% (19/166 individuals; minor allele frequency of 0.06) of our case-control study from Moshi, Tanzania (Table 2). The 1000 Genomes Project reports an overall minor allele frequency of 0.01 and 0.05 for individuals from West Africa [33]. All individuals from Tanzania in our study were of African descent, while 39 of the 50 newborns carrying the variant allele were from mothers who self-reported as African American (Fisher’s exact test, *p* < 0.0001 comparing African descent to non-African descent). These results indicate that this polymorphism may be significantly more prevalent in individuals with African heritage. However, the polymorphism was not enriched among individuals with cervical intraepithelial neoplasia (CIN; Fisher’s exact test, *p* = 0.62) or invasive cervical cancer (ICC; *p* = 0.39). In addition, pyrosequencing of peripheral blood DNA from all nine individuals with CIN or ICC showed the presence of the polymorphism. We confirmed the polymorphism by Sanger sequencing of biopsy specimens for two patients with ICC and two patients with CIN; peripheral blood DNA from one patient with ICC and one with CIN was also sequenced with concordant results (data not shown). These results suggest that the presence of this polymorphism may not be associated with the onset or progression of cervical dysplasia, though larger studies are required for confirmation.

**Figure 3:**
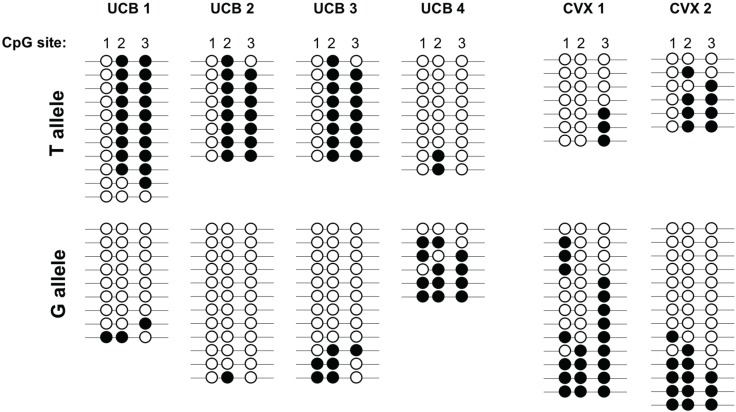
The variant allele lacks methylation at CpG position 1 of the *IGF2* DMR. Bisulfite sequencing of cloned alleles for four umbilical cord blood DNA specimens (UCB1-UCB4) and two cervical biopsy specimens (CVX1-CVX2) from individuals carrying the variant allele. Each line represents a given clone and each circle represents one of the CpG dinucleotides within the *IGF2* DMR. Filled circles indicate that the cytosine was methylated and the unfilled circles indicate that the cytosine was unmethylated within that clone. The data indicate that the variant T allele exhibits a lack of methylation at the first CpG site within the *IGF2* DMR.

To confirm that the presence of the polymorphism influences the methylation status of CpG position 1 within the *IGF2* DMR, we performed bisulfite sequencing of cloned alleles for six individuals carrying the variant allele (Figure 3). All six individuals showed a lack of methylation at CpG position 1 on the variant T allele using this method. For three of the four cord blood specimens (UCB1-3 in Figure 3), the variant T allele was likely the paternally derived allele, since the other two CpG positions were nearly fully methylated across the clones analyzed. We are able to infer this because this region is normally differentially methylated, with methylation present on the paternally-derived allele [34,35]. For the two cervical biopsy specimens analyzed, more generalized hypomethylation at this DMR, a common finding in other gynecologic cancers [12,36], made it difficult to determine the parental origin of each allele. Regardless, these results demonstrate that the presence of the polymorphism prevents methylation at CpG position 1 by changing the CG DNA methyltransferase target sequence on the WT allele to a CC dinucleotide combination not recognized by these enzymes. Furthermore, if this polymorphism is present on the paternally-derived chromosome, this theoretically reduces the overall methylation at this locus from ~50% to ~34% in a diploid cell.

**Table 1:** Newborn Epigenetics Study.

rs116779517	WT (%)	Heterozygote (%)
Caucasian	510 (99)	7 (1)
African American	445 (92)	39 (8)
Asian/Pacific Islander	18 (100)	0 (0)
Native American	4 (100)	0 (0)
Multiracial	10 (100)	0 (0)
Other	53 (96)	2 (4)
Don't Know	5 (100)	0 (0)
Missing	9 (82)	2 (18)
**Total**	**1054 (95)**	**50 (5)**

**Table 2:** Tanzania Cervical Cancer Study.

rs116779517	WT (%)	Heterozygote (%)
Non-neoplastic	88 (90)	10 (10)
Neoplastic	59 (87)	9 (13)
**Total**	**147 (89)**	**19 (11)**

## 4. Discussion

The presence of this polymorphism is crucial to interpretation of any methylation studies of the *IGF2* DMR, especially since the most common abnormality reported for this DMR in the literature is hypomethylation. The presence of this variant allele could go unnoticed using other bisulfite-based methods and lead to a finding of hypomethylation, assumed to be due to environmentally driven epigenetic variability rather than genetic variation. Indeed, we excluded the individuals carrying this variant in our own studies because these samples failed the pyrosequencing runs.

It has been known for some time that genetic and epigenetic variation together influence gene expression and disease susceptibility, however, specific empirical data are limited. Within the *IGF2/H19* imprinted domain, Tobi *et al.* have reported that SNP rs2239681, upstream of the *IGF2* DMR, is associated with decreased methylation [23], but this SNP exerts this effect over distance and does not directly alter CpG sites. A single nucleotide variant that alters methylation at a CpG site within one of the core binding sites for the CTCF insulator protein that regulates the *IGF2/H19* imprinted domain has also been identified. The presence of the variant allele (rs10732516) affects genetically-induced hypomethylation, which is associated with higher birth weight [21]. In another study, Oertel *et al.* identified an A>G SNP (rs1799971) that introduces a new CpG site in the *OPRM1* gene; methylation at this site reduces expression and opioid receptor signaling [37]. Thus although an increasing number of studies are examining the *cis* activity of SNPs in relation to DNA methylation, there are also SNPs that directly affect methylation changes with a consequent change in phenotype that should also be further investigated.

Genetically predetermined hypomethylation may have substantial relevance to the expression of *IGF2* and risk of disease, perhaps more so than any environmentally-induced variation. We previously reported that a 1% change in methylation at this DMR was associated with a two-fold change in *IGF2* transcription [18]. Others have reported that hypomethylation of these same CpGs is associated not only with loss of *IGF2* imprinting, but also to risk of colon cancer, with hypomethylation detected in 10% of an otherwise unaffected population [24,25,38]. Race was not reported in these studies so it is unclear how this polymorphism might have contributed to the hypomethylation reported. Furthermore, the relevance of loss of imprinting is also uncertain, since other studies have detected loss of imprinting primarily where overall *IGF2* expression is low [12,39]. We did not detect an association between the presence of the polymorphism and cervical neoplasia or cancer, however we examined a relatively small number of individuals. Nevertheless, if the prevalence of this polymorphic variant in individuals of African descent is confirmed by others, these findings could have substantial implications for colon cancer risk assessment in these individuals. Hypomethylation at this DMR may increase risk of cancer regardless of whether the hypomethylation is genetically and/or epigenetically determined.

We did not determine the parental origin of the rare variant allele. However, we have previously found that these CpGs are fully methylated in mature human spermatozoa [35], confirming reports of others [34,40] and consistent with the methylation in somatic tissues being on the paternally-derived allele. Thus if the G>C polymorphism is inherited from the father, it might be expected that there would be no methylation present at this CpG site. However, we did not observe 0% methylation at this CpG site in any of the samples analyzed that carry the nucleotide variant. The reason for this is evident when tracking the order of nucleotide incorporation in the pyrosequencing “sequence to analyze” and taking into account the pattern of methylation expected on the paternal allele. The sequence variant leads to additional “T” nucleotides incorporated at the first CpG site (*i.e.*, the cytosine cannot be methylated in CpC context so both are converted by bisulfite, adding two Ts); when the C is added for incorporation for the first CpG site, it is actually the second CpG site C nucleotide that ends up being incorporated at this position for the variant allele. Thus methylation at the first CpG site actually reflects that at the second CpG site. The second CpG site in turn exhibits very low methylation.

## 5. Conclusions

In conclusion, through bisulfite pyrosequencing and subsequent confirmation by nucleotide sequencing, we identified and validated a polymorphic variant that directly prevents methylation at one of the CpG dinucleotides that comprise the *IGF2* DMR, a region for which hypomethylation has previously been associated with a compromised environment during prenatal development as well as risk of colon cancer. We found the variant in two distinct populations including one from Tanzania, Africa and the other from Durham, NC, USA. The variant is significantly more common in, but may not be exclusive to, individuals of African descent, and these findings indicate that epigenetic analysis of this region should take into account the potential genetic contribution to hypomethylation.
